# Surgical Site Infection Prevention Research: A Bibliometric Analysis of the Top 50 Highly Cited Articles

**DOI:** 10.7759/cureus.101566

**Published:** 2026-01-14

**Authors:** Lora H Alquzi, Nawaf M Alharbi, Sami K Alghamdi, Waleed K Alotaibi, Abdullah D Alessimii, Raghad H Algaed, Majed F Alasmi, Nouf K Yunus, Lujain S Alshamrani, Mohammed K Alburghash

**Affiliations:** 1 Department of Surgery, College of Medicine, Umm Al-Qura University, Al-Qunfudhah, SAU; 2 Department of Surgery, College of Medicine, Al-Rayan Colleges, Madinah, SAU; 3 Department of Surgery, College of Medicine, Imam Abdulrahman Bin Faisal University, Dammam, SAU; 4 Department of Surgery, College of Medicine, Umm Al-Qura University, Makkah, SAU; 5 Department of Surgery, College of Medicine, Cairo University, Cairo, EGY; 6 Department of Surgery, College of Medicine, Al-Jouf University, Sakaka, SAU; 7 Department of Surgery, College of Medicine, Imam Mohammad Ibn Saud Islamic University, Riyadh, SAU; 8 Department of Surgery, College of Medicine, University of Tabuk, Tabuk, SAU; 9 Department of Surgery, College of Medicine, King Faisal University, Al-Ahsa, SAU; 10 Department of General Surgery, King Fahad Specialty Hospital, Tabuk, SAU

**Keywords:** antibiotic prophylaxis, bibliometric analysis, perioperative infection control, surgical site infection, surgical site infection prevention

## Abstract

Surgical site infections remain a major concern in modern surgical practice, as they are among the most frequent postoperative complications and place a substantial burden on patients and healthcare systems worldwide. Through a bibliometric analysis, this study examined the 50 most highly cited publications addressing surgical site infection prevention, aiming to describe research trends, dominant study designs, involved specialties, and the overall strength of the available evidence. A comprehensive search of the Web of Science Core Collection was performed in March 2025 using predefined terms related to surgical site infection and preventive strategies. Following the screening of 2,728 records, the top 50 most cited articles were selected for analysis. Bibliographic characteristics and study-level variables, including study design, level of evidence, specialty, and reported outcomes, were independently extracted by two reviewers, and descriptive analyses were conducted. The included publications demonstrated a predominance of retrospective cohort studies, with most articles classified as level two evidence. General surgery and orthopedic surgery were the most frequently represented specialties. Research output peaked during the 2010s, a period that included a higher proportion of controlled trials but also underscored the ongoing challenges of conducting large-scale interventional studies. Overall, this analysis highlights substantial scholarly interest in surgical site infection prevention while revealing a continued shortage of randomized studies despite a sustained global attention to this topic.

## Introduction and background

Surgical site infections (SSIs) remain a common and clinically significant complication following surgical procedures worldwide. They are associated with prolonged recovery, increased postoperative morbidity, higher readmission rates, and substantial healthcare costs [1]. SSIs occur when microorganisms contaminate the surgical wound during or after an operation, and their development is influenced by multiple factors, including patient-related characteristics, the type and complexity of surgery, wound classification, and adherence to preventive measures [2].

Despite advances in surgical techniques and infection prevention strategies, SSIs continue to be reported across a wide range of healthcare settings. Interventions such as timely antibiotic prophylaxis, appropriate skin antisepsis, and the implementation of perioperative care bundles have demonstrated effectiveness in reducing infection rates [3,4]. However, the adoption and consistent application of these measures vary considerably between institutions and regions, contributing to ongoing differences in SSI incidence worldwide. Operative factors such as prolonged surgical duration and perioperative blood transfusion have also been shown to increase the risk of postoperative infections [5,6].

Bibliometric analysis provides a systematic approach to evaluating the development and impact of research within a specific field. By examining the most highly cited publications, bibliometric studies are able to identify articles that are “influential” in terms of citation impact (i.e., those receiving the highest total citation counts), as well as dominant research themes and existing knowledge gaps.

In this review, “influential articles” were defined as those with the highest total citation counts, reflecting their impact on subsequent SSI prevention research.

In this context, the present bibliometric review identifies and analyzes the 50 most highly cited articles on SSI prevention. The study aims to: (1) describe citation patterns and publication trends; (2) identify influential authors, journals, institutions, and countries; (3) characterize the major research themes and keywords; and (4) highlight the existing gaps to guide future SSI prevention research.

## Review

Methodology 

This study followed standard bibliometric review methodology, incorporating systematic database searching, predefined eligibility criteria, citation-based ranking, structured data extraction, and descriptive analysis of publication and citation patterns.

This bibliometric analysis aimed to identify the 50 most-cited publications addressing SSI prevention. A comprehensive search of the Web of Science Core Collection was conducted in March 2025 using predefined terms related to surgical site infections, infection prevention strategies, perioperative infection control, and antibiotic prophylaxis. Web of Science was selected as the primary database due to its comprehensive citation indexing and suitability for bibliometric analyses. Although PubMed is a highly valuable resource for biomedical literature retrieval, it does not provide complete citation data required for accurately ranking highly cited publications, which was essential for the objectives of this study. Therefore, using Web of Science allowed us to apply a consistent and objective citation-based ranking of SSI prevention articles that aligned directly with the aims of this bibliometric review. The initial search yielded 2,728 records.

**Figure 1:**
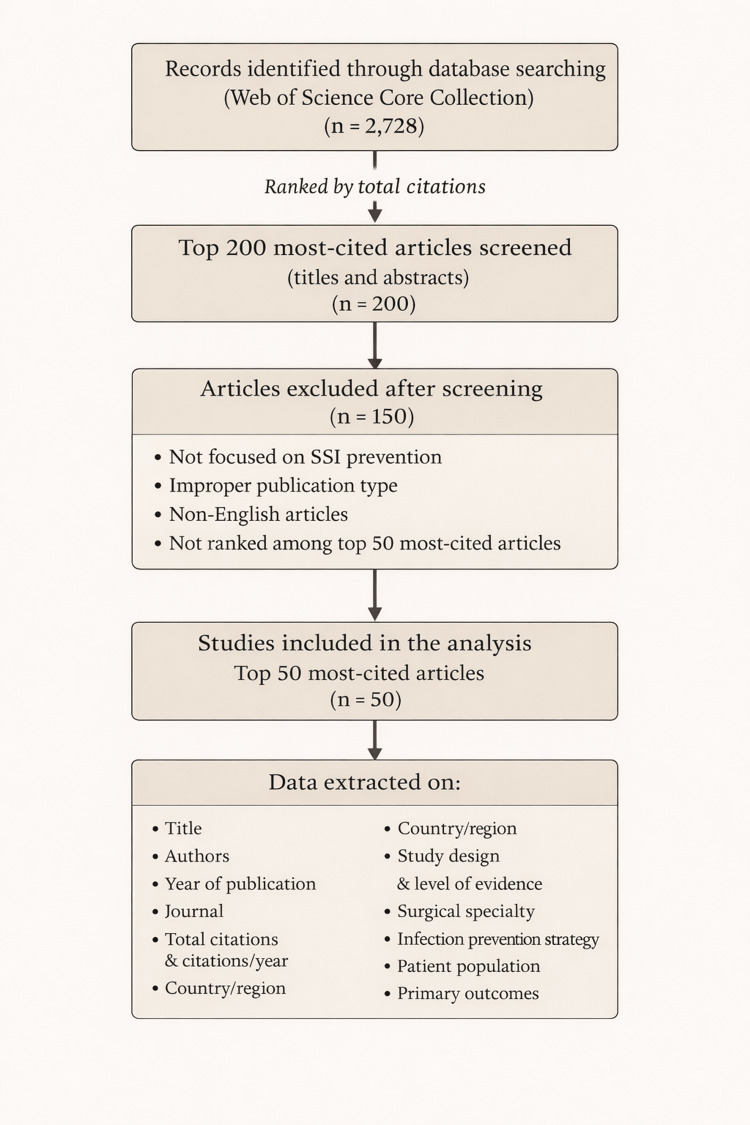
Summary Flowchart of the Methodology Records were retrieved from the Web of Science Core Collection (n=2,728) and ranked according to total citation counts. To focus on the most influential literature, the top 200 most-cited articles were screened based on titles and abstracts and assessed for eligibility. Articles were excluded due to lack of relevance to surgical site infection prevention, inappropriate publication type, non-English language, or lower citation ranking after full-text assessment. The final 50 most-cited studies were included in the analysis, and bibliometric and study-level data were extracted.

All retrieved records were ranked according to total citation count. Screening was performed in sequential stages, with titles and abstracts reviewed to identify studies primarily focused on SSI prevention. Full-text assessment was conducted for potentially eligible articles. Studies were included if they were published in peer-reviewed journals, involved human subjects, and evaluated strategies intended to reduce SSIs. Articles focusing exclusively on antimicrobial resistance, non-human studies, abstracts, editorials, conference proceedings, and non-English publications were excluded. The detailed screening and selection process is summarized in Figure 1.

In line with common practices in top-cited bibliometric analyses, we limited our evaluation to the 50 most highly cited articles. This threshold was selected to allow detailed manual review, accurate data extraction, and meaningful comparison across studies, while still capturing landmark publications that have shaped the field of SSI prevention.

Two reviewers independently screened and assessed all eligible studies and disagreements were resolved through discussion and consensus. For each included article, data were extracted on publication year, journal, total citation count, average citations per year, country or region of origin, study design, level of evidence, surgical specialty, infection prevention methods, patient population, and primary outcomes. All extracted data were manually cross-checked for accuracy. Descriptive statistical analyses were performed using Microsoft Excel (Microsoft, Redmond, WA, USA) and verified using IBM SPSS Statistics (IBM Corp., Armonk, NY, USA).

This study represents a multi-institutional academic collaboration in which authors from different institutions contributed complementary expertise in bibliometric analysis, study design, data extraction, and manuscript preparation. Such collaboration aimed to enhance methodological rigor and ensure comprehensive interpretation of the findings.

In accordance with the established bibliometric standards, the analysis was performed in two complementary phases. First, a performance analysis was conducted to describe publication productivity, authorship patterns, journal distribution, and citation impact. Second, science-mapping techniques were applied to explore conceptual relationships using keyword co-occurrence networks and thematic clustering, allowing visualization of how research topics in SSI prevention have evolved over time.

Science-mapping analysis was performed using VOSviewer software (developed by Nees Jan van Eck and Ludo Waltman at the Centre for Science and Technology Studies (CWTS), Leiden University, Leiden, The Netherlands), which constructs co-occurrence networks and visualizes keyword clusters based on bibliometric relationships.

Results

The findings are presented in two parts: (1) a performance analysis describing publication characteristics and citation impact and (2) science-mapping visualizations illustrating conceptual relationships and thematic trends.

A total of 50 studies met the eligibility criteria and were included in this bibliometric review following the predefined screening process. The distribution of publications and total citation counts across journals is summarized in Table 1.

**Table 1:** Publication count and total citations per journal This table summarizes the distribution of the top 50 most-cited surgical site infection prevention articles across journals. For each journal, the number of included publications and the corresponding total citation counts are presented, reflecting the relative contribution and citation impact of each journal within the SSI prevention literature.

Journal Name	Count	Total Citations Per Journal
American Journal of Infection Control	4	148
Journal of Hospital Infection	4	156
Diseases of The Colon & Rectum	2	44
Global Spine Journal	2	65
Joint Commission Journal on Quality and Patient Safety	2	66
Journal of Bone and Joint Surgery	1	75
Journal of the American College of Surgeons	2	108
Obstetrics & Gynecology	2	78
Surgical Infections	1	54
Annals of Surgery	1	233
Antimicrobial Resistance and Infection Control	1	61
American Journal of Perinatology	1	16
American Society for Microbiology	1	52
Antibiotics	1	15
AORN Journal	1	34
Arquivos Brasileiros de Cardiologia	1	24
BMC Health Services Research	1	28
Canadian Journal of Surgery	1	50
Clinics in Colon and Rectal Surgery (Thieme)	1	32
Cureus	1	19
European Journal of Cardiothoracic Surgery	1	24
Infection Control and Hospital Epidemiology	1	35
International Journal of Infectious Diseases	1	29
International Journal of Surgery	1	13
JAMA - *Journal of the American Medical Association*	1	180
Journal of Antimicrobial Chemotherapy	1	84
Journal of Gastroenterology and Hepatology	1	47
Journal of the Royal Colleges of Surgeons of Edinburgh and Royal College of Surgeons in Ireland	1	73
Lippincott Williams & Wilkins (*Spine Journal*)	1	13
Open Forum Infectious Diseases	1	14
Orthopaedic Nursing	1	20
Orthopedics	1	25
Patient Safety in Surgery	1	20
PLoS One	1	25
Surgery	1	19
The Surgical Clinics of North America	1	46
World Journal of Emergency Surgery	1	14

The key characteristics and primary outcomes of the included studies are summarized in Table 2.

**Table 2:** Key information and primary outcomes of top-cited surgical site infection prevention studies This table summarizes the key characteristics and primary outcomes of the top 50 most-cited surgical site infection prevention studies. For each included study, information is presented on citation metrics (total citations and average citations per year), study title, and the primary clinical outcomes evaluated, including surgical site infection rates, mortality, morbidity, and readmission where applicable.

Title	Complete number of citations	Average number of citations annually	Primary outcomes
Surgical site infection prevention Time to move beyond the surgical care improvement program [5]	233	16.64	SSI rates
Surgical site infection prevention: The importance of operative duration and blood transfusion - Results of the First American College of Surgeons - National Surgical Quality Improvement Program Best Practices Initiative [6]	189	11.11	SSI rates, mortality and morbidity
Surgical site infection prevention: A review [2]	180	90	SSI rates
The role of topical antibiotics used as prophylaxis in surgical site infection prevention [7]	84	6	SSI rates
Surgical site infection prevention and control: An emerging paradigm [8]	75	4.68	SSI rates, readmission, mortality
Laminar airflow and the prevention of surgical site infection. More harm than good? [9]	73	7.3	SSI rates
*Staphylococcus aureus* and surgical site infections: benefits of screening and decolonization before surgery [10]	69	7.66	SSI rates
Patient engagement with surgical site infection prevention: an expert panel perspective [11]	61	4.69	SSI rates
Evidence that a regional surgical collaborative can transform care: Surgical site infection prevention practices for colectomy in Michigan [12]	54	1.74	SSI rates
Surgical site infection prevention: How we do it [13]	54	2.16	SSI rates
Influences on compliance with standard precautions among operating room nurses [14]	54	1.2	SSI rates
Perioperative antibiotic prophylaxis in total joint arthroplasty: A single dose is as effective as multiple doses [15]	52	8.66	SSI rates
No concordance with surgical site infection prevention guidelines and rates of surgical site infections for general surgical, neurological, and orthopedic procedures [16]	52	3.71	SSI rates
Prevention of deep sternal wound infection in cardiac surgery: a literature review [17]	51	7.28	SSI rates
Factors influencing antibiotic prophylaxis for surgical site infection prevention in general surgery: a review of the literature [18]	50	3.12	SSI rates
Perioperative infection control and its effectiveness in hepatectomy patients [19]	47	2.61	SSI rates
Prophylactic antibiotics and prevention of surgical site infections [20]	46	4.6	SSI rates
Outcomes associated with a five-point surgical site infection prevention bundle in women undergoing surgery for ovarian cancer [21]	41	5.12	SSI rates, readmission rates
Decreased surgical site infection rate in hysterectomy: Effect of a gynecology-specific bundle [22]	37	5.28	SSI rates, length of hospital stay and 30-day postoperative readmission rate
Surgical site infection prevention following spine surgery [23]	36	7.2	SSI rates
Surgical site infection prevention following total hip arthroplasty in Australia: A cost-effectiveness analysis [24]	36	3	SSI rates and economic impact
Reducing surgical site infections at a pediatric academic medical center [25]	36	2.25	SSI rates and morbidity
Colorectal bundles for surgical site infection prevention: A systematic review and meta-analysis [26]	35	7	SSI rates, morbidity and economic impact
Perioperative strategies for surgical site infection prevention [27]	34	4.86	SSI rates
Surgical site infection prevention initiative: Patient attitude and compliance [28]	33	2.2	SSI rates
Surgical site infection: The clinical and economic impact [29]	32	5.33	SSI rates, morbidity, mortality, readmission and economic impact
Reducing surgical complications [30]	30	1.66	SSI rates
Implementation of surgical site infection surveillance in low- and middle-income countries: A position statement for the International Society for Infectious Diseases [31]	29	5.8	SSI rates, morbidity, mortality, and economic impact
Surgical site infections in spine surgery: Preoperative prevention strategies to minimize risk [32]	29	4.14	SSI rates
Implementation interventions in preventing surgical site infections in abdominal surgery: A systematic review [33]	28	5.6	SSI rates
Quality appraisal of clinical guidelines for surgical site infection prevention: A systematic review [34]	25	3.5	Readmissions, or mortality associated with SSI
An Effective Bundled Approach Reduces Surgical Site Infections in a High-Outlier Colorectal Unit [35]	25	3.5	SSI rates
Evidence-Based Update to the U.S. Centers for Disease Control and Prevention and Healthcare Infection Control Practices Advisory Committee Guideline for the Prevention of Surgical Site Infection: Developmental Process [36]	25	2.7	SSI rates
Patient experience with mupirocin or povidone-iodine nasal decolonization [37]	25	2.27	SSI rates
Compliance with surgical antibiotic prophylaxis at an Australian teaching hospital [38]	25	1.92	SSI rates, antimicrobial resistance
Surgical site infection prevention bundle in cardiac surgery [39]	24	4	SSI risk factors
Sternal surgical site infection prevention - is there any room for improvement? [40]	24	1.72	SSI rates
Surgical site infection prevention: a global priority [41]	21	2.33	SSI rates, SSI risk factors, effectiveness of prevention strategies, readmissions
Surgical site infection and pathogens in Ethiopia: a systematic review and meta-analysis [42]	20	4	Prevalence of SSI and most common pathogens
Clinical practice guideline surgical site infection prevention [43]	20	1.66	SSI rates, morbidity, mortality and readmission
Antibiotic prophylaxis in surgery: Current insights and future directions for surgical site infection prevention [44]	19	9.5	SSI rates, minimizing antibiotic-related risks
Perioperative oral care can prevent surgical site infection after colorectal cancer surgery: A multicenter, retrospective study of 1,926 cases analyzed by propensity score matching [45]	19	6.33	SSI rates
Implementation of surgical quality improvement: auditing tool for surgical site infection prevention practices [46]	19	1.9	SSI rates, identification of defects in infection prevention practices
A multifaceted surgical site infection prevention bundle for cesarean delivery [47]	16	4	SSI rates
Meta-analysis of clinical trials comparing cefazolin to cefuroxime, ceftriaxone, and cefamandole for surgical site infection prevention [48]	15	5	SSI rates
Surgical site infection prevention bundle in elective colorectal surgery [49]	15	5	SSI rates
Cefazolin vs second-line antibiotics for surgical site infection prevention after total joint arthroplasty among patients with a beta-lactam allergy [50]	14	7	SSI rates, interoperative hypersensitivity reactions (HSRs) as a secondary outcome
Surgical site infection prevention and management in immunocompromised patients: A systematic review of the literature [51]	14	3.5	SSI rates
Surgical site infection prevention through bundled interventions in hip replacement surgery: A systematic review [52]	13	3.25	SSI rates
Surgical site infection following neuromuscular posterior spinal fusion fell 72% after adopting the 2013 best practice guidelines [53]	13	3.25	incidence of SSI within one year of surgery

Study design

**Figure 2:**
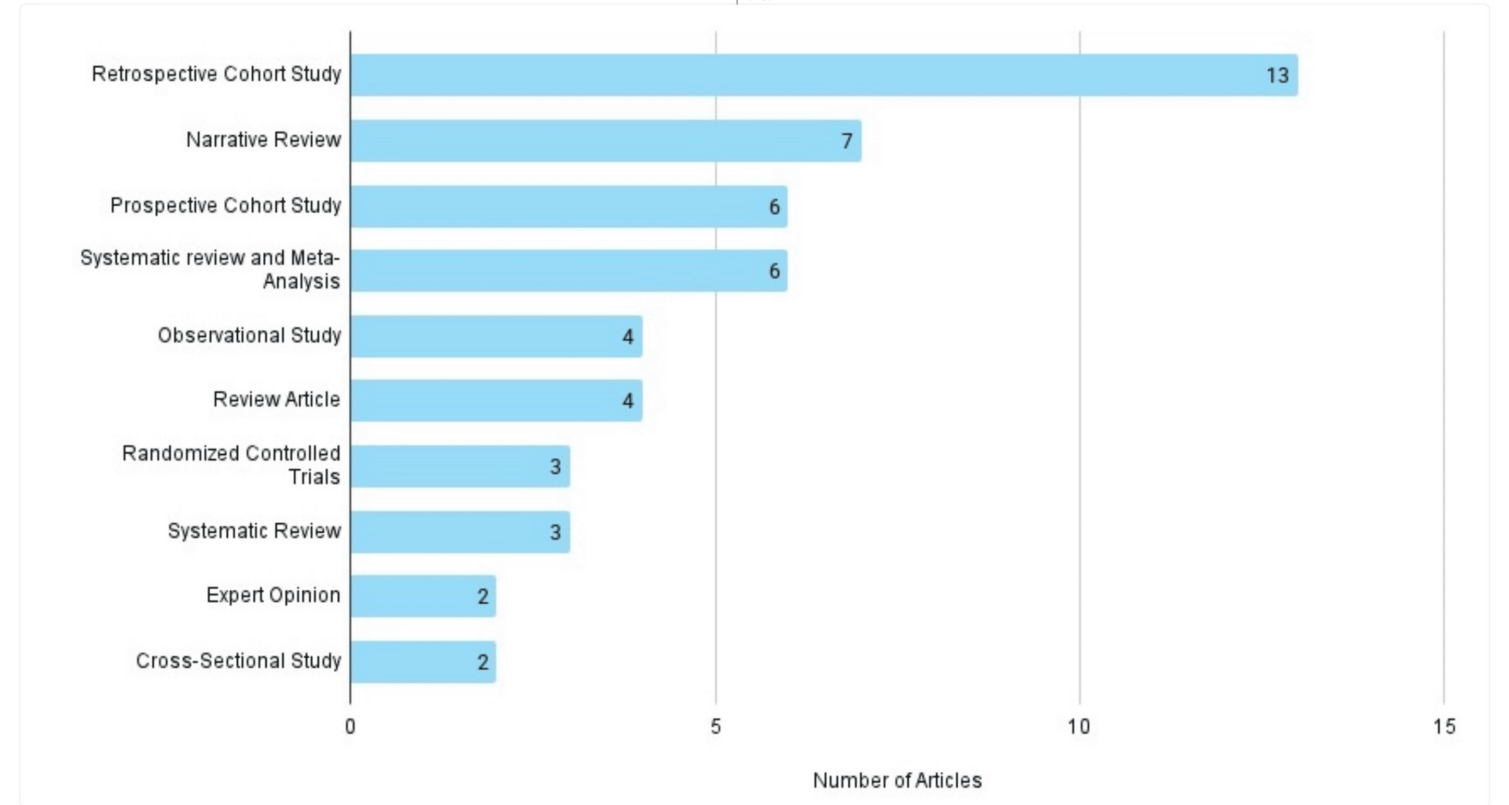
Study Designs of the Top 50 Most-Cited SSI Prevention Articles This figure presents the frequency of different study designs represented among the included publications. The horizontal bars indicate the number of studies corresponding to each study design category.

An analysis of the study designs demonstrated that retrospective cohort studies constituted the largest proportion of the highly cited literature. This was followed by narrative reviews, prospective cohort studies, and systematic reviews with or without meta-analysis. Randomized controlled trials represented a smaller fraction of the included studies. The distribution of study designs among the 50 most-cited articles is illustrated in Figure 2.

Level of evidence

**Figure 3:**
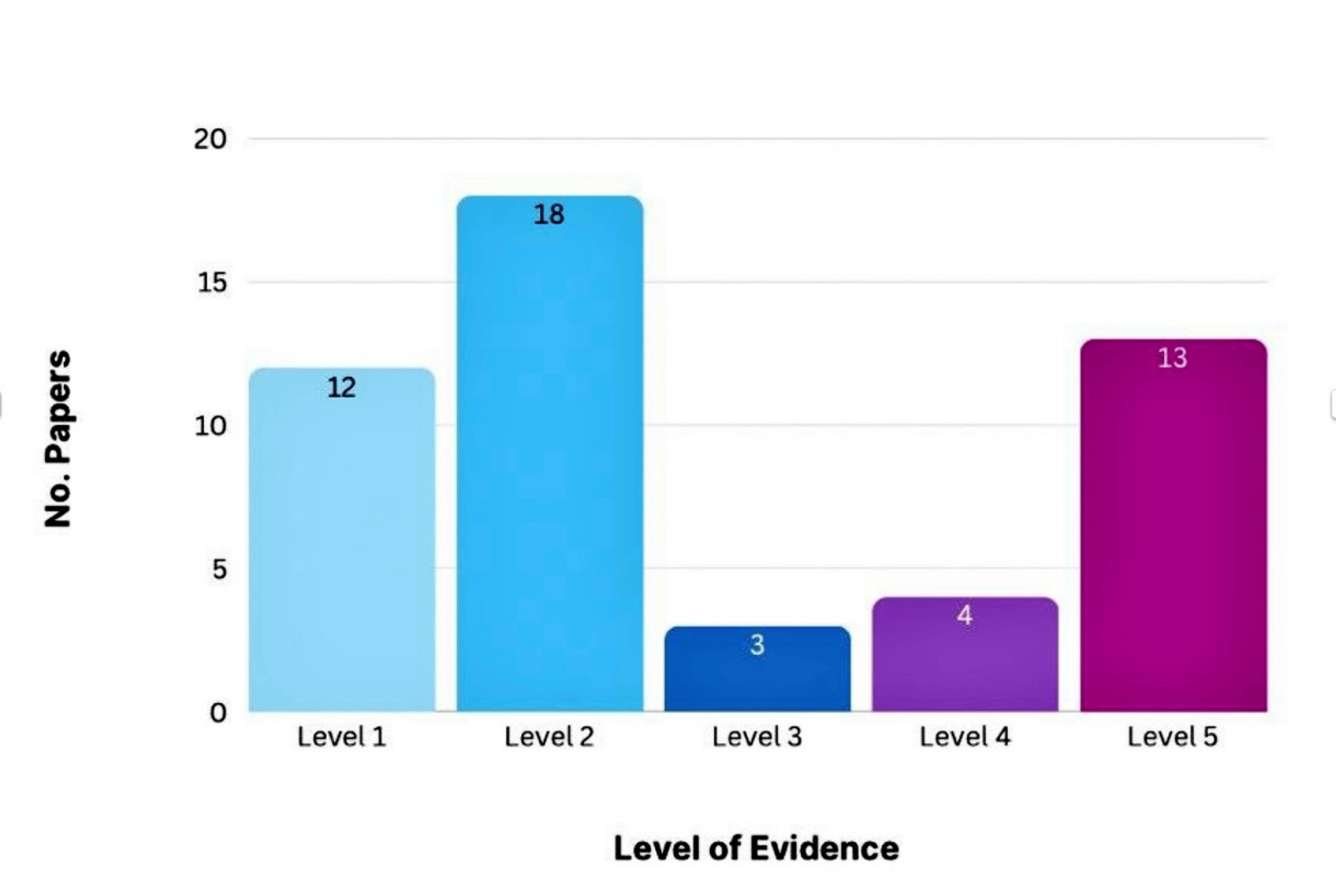
Levels of Evidence of the Top 50 Most-Cited SSI Prevention Articles This figure illustrates the distribution of included studies according to their level of evidence. The vertical bars represent the number of publications corresponding to each evidence level.

Most of the included articles were classified as level II evidence, indicating a predominance of moderate-quality observational research among influential SSI prevention studies. Smaller proportions of studies represented level I evidence or lower levels of evidence, reflecting methodological diversity within the highly cited literature. The distribution of levels of evidence among the included studies is shown in Figure 3.

Temporal distribution

**Figure 4:**
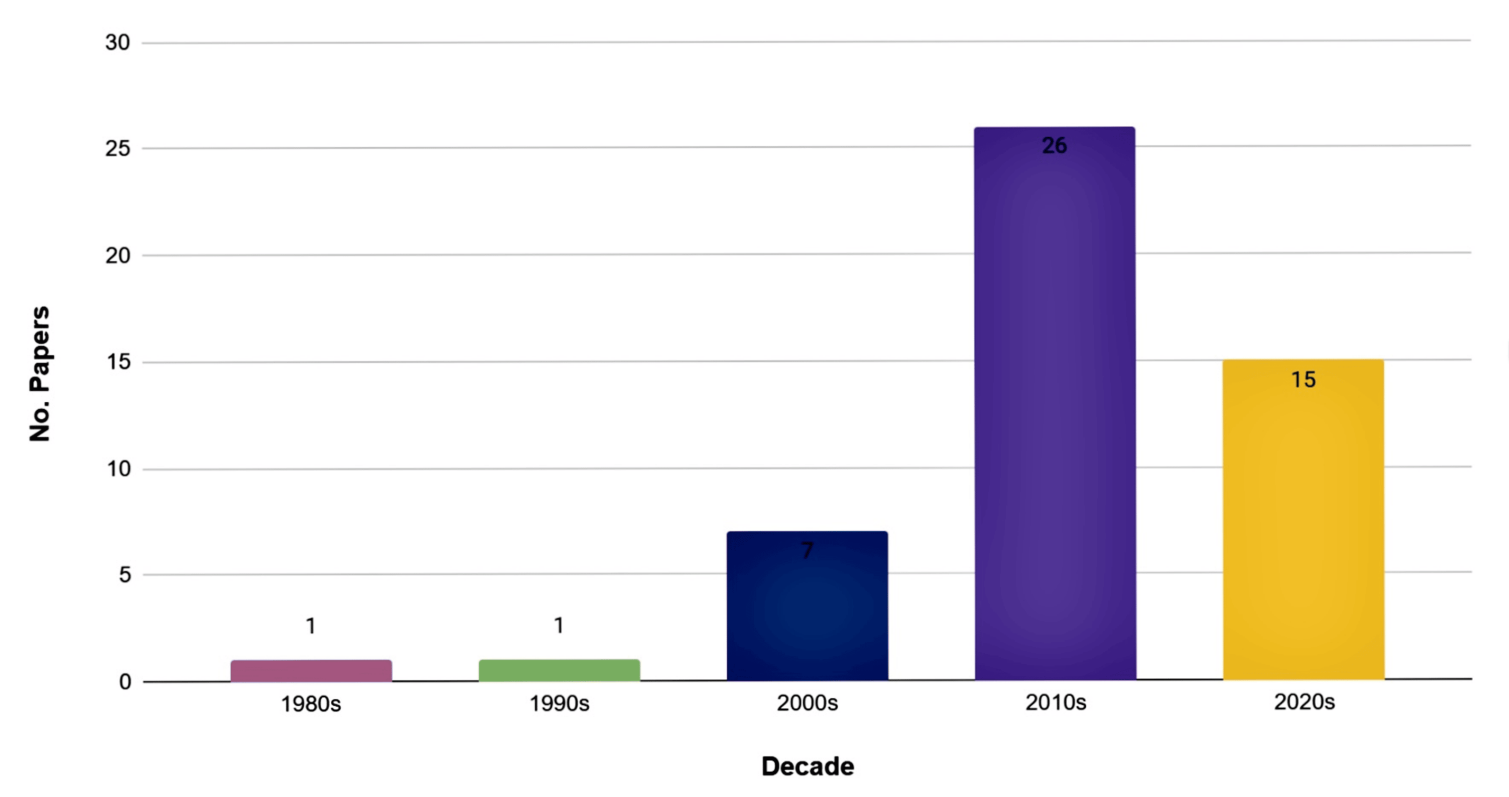
Articles per Decade Among the Top 50 Most-Cited SSI Prevention Studies This figure displays the distribution of included articles according to the decade of publication. The vertical bars represent the number of studies published within each decade.

An evaluation of publication trends revealed a marked increase in influential publications during the 2010s, followed by continued output in the early 2020s. In contrast, earlier decades contributed relatively few highly cited articles. The temporal distribution of the included publications by decade is summarized in Figure 4.

Surgical specialties

**Figure 5:**
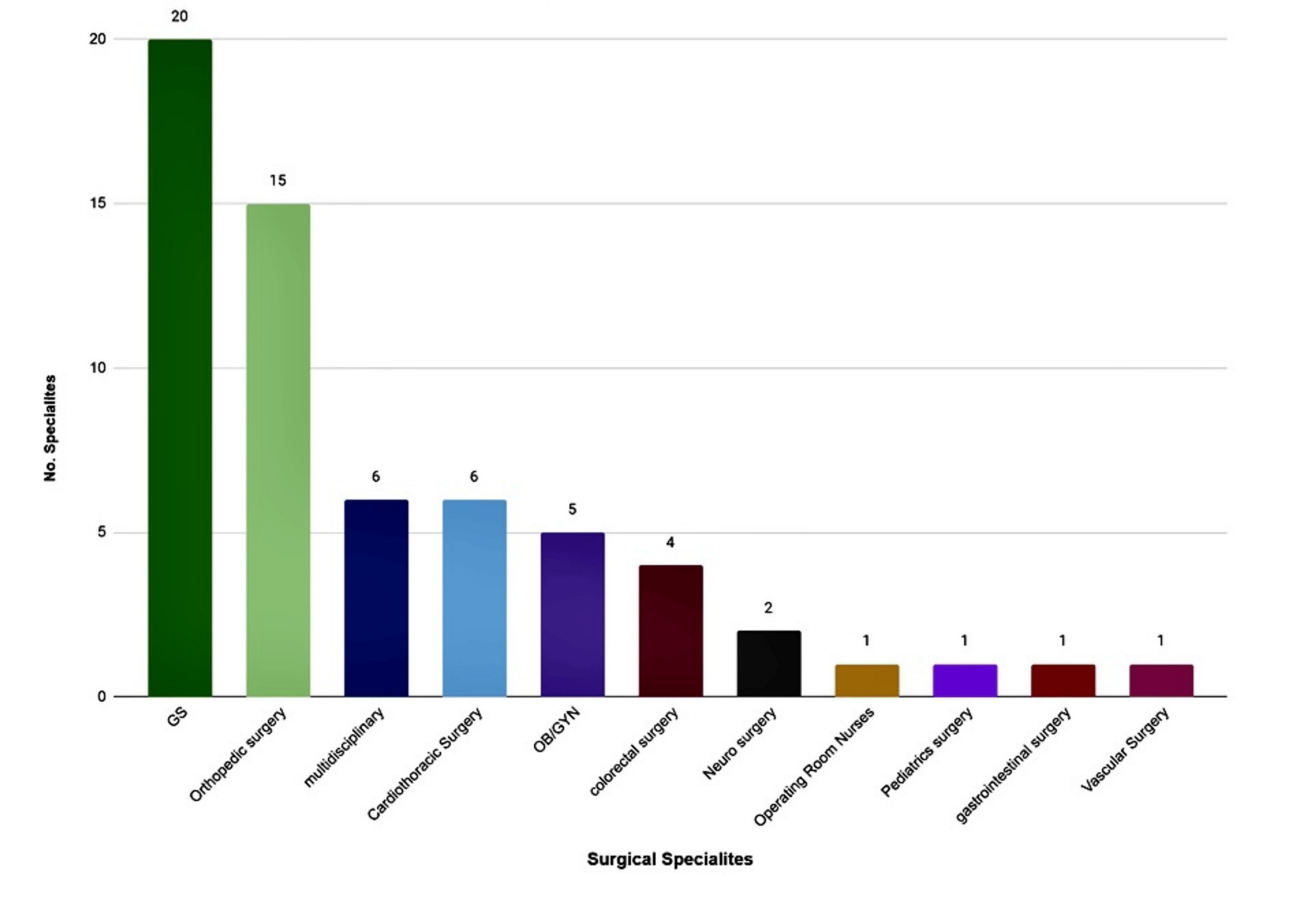
Surgical Specialties Represented in the Top 50 Most-Cited SSI Prevention Articles This figure illustrates the distribution of included studies across different surgical specialties. The vertical bars represent the number of publications corresponding to each specialty. Abbreviation: SSI=Surgical site infection; GS=general surgery.

Regarding surgical specialty, general surgery accounted for the highest number of included articles, followed by orthopedic surgery. Other specialties, including multidisciplinary surgery, cardiothoracic surgery, obstetrics and gynecology, colorectal surgery, and neurosurgery, were less frequently represented. The distribution of surgical specialties across the 50 most-cited articles is presented in Figure 5.

Keyword analysis

**Figure 6:**
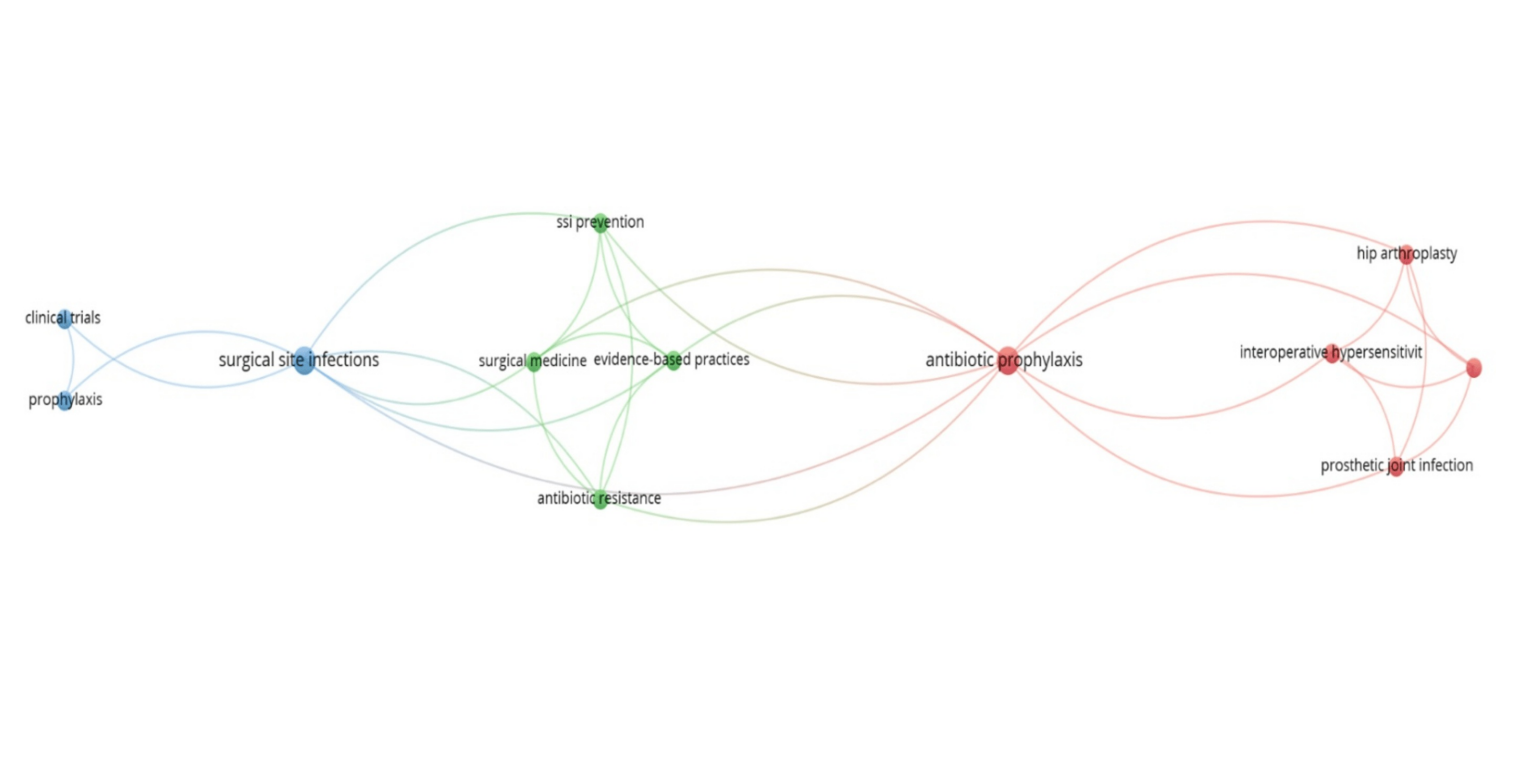
Keyword Co-Occurrence Network Map This network map visualizes the co-occurrence of author keywords within the included studies. Each node represents a keyword, with node size proportional to its frequency of occurrence. Links between nodes indicate co-occurrence relationships, and link thickness reflects the strength of these associations. Colors denote clusters generated automatically by the mapping algorithm, grouping keywords that frequently appear together. In our dataset, these clusters broadly reflected different thematic areas: blue (clinical research concepts), green (evidence-based practice and prevention themes), and red (perioperative and procedure-related topics). The network was generated using VOSviewer, and clusters were formed automatically based on co-occurrence relationships.

Keyword co-occurrence analysis demonstrated that terms related to antibiotic prophylaxis, surgical site infections, evidence-based practice, and prosthetic joint infection were among the most frequently interconnected concepts. These relationships highlight the dominant research themes and conceptual interconnections within the SSI prevention literature. The keyword co-occurrence network map is shown in Figure 6.

In the keyword co-occurrence analysis, node size represented the frequency of each keyword, while node colors indicated distinct thematic clusters generated by the mapping algorithm. Three main clusters were observed, reflecting clinical research concepts, evidence-based practice themes, and perioperative or procedure-related topics, respectively.

Among these, “surgical site infection / SSI” appeared as the most frequently occurring keyword, followed by “antibiotic prophylaxis” and “evidence-based practice.”

Discussion

This bibliometric analysis highlights several key patterns in SSI prevention research. The majority of influential publications were concentrated in high-impact surgical and infectious disease journals, originated primarily from North America and Europe, and were largely composed of observational studies rather than randomized trials. These findings suggest that although SSI prevention has attracted sustained academic attention, much of the influential evidence continues to arise from non-experimental research designs, indicating opportunities for stronger prospective and interventional studies in the future.

A notable finding was the high proportion of level V evidence among the most-cited publications. This pattern likely reflects the influential role of expert consensus statements, guidelines, and large observational studies in shaping SSI prevention practices. Although these studies are lower in the evidence hierarchy, they frequently inform clinical protocols, which explains their high citation impact.

SSIs remain a major contributor to postoperative morbidity, prolonged hospitalization, and increased healthcare costs worldwide despite continued advances in surgical techniques and infection-control practices. This bibliometric analysis provides insight into the studies that have most strongly influenced SSI prevention research over recent decades [8-12].

One of the most notable findings was the predominance of retrospective cohort studies among the most highly cited publications. Although randomized controlled trials provide stronger causal inference, their limited representation reflects the logistical, ethical, and practical challenges associated with conducting large-scale interventional studies in surgical settings [13-17]. Nevertheless, observational research has played a pivotal role in shaping SSI prevention strategies, informing clinical guidelines, and guiding perioperative practice [18-20].

The concentration of highly cited publications during the 2010s aligns with the global expansion of patient safety initiatives, standardized SSI prevention bundles, and quality-improvement programs [21-24]. This period coincided with heightened awareness of healthcare-associated infections and increased emphasis on evidence-based perioperative care. Continued publication activity in the 2020s suggests sustained scholarly interest in SSI prevention, although newer studies may be disadvantaged in citation-based analyses due to shorter exposure time [25-27].

General surgery and orthopedic surgery accounted for the majority of influential publications, likely reflecting their high procedural volume and historically elevated SSI risk [28-30]. In contrast, several surgical subspecialties were underrepresented among the most-cited literature. This finding suggests potential gaps in specialty-specific SSI prevention research and highlights opportunities for future investigations tailored to distinct surgical populations [31-33].

Keyword analysis further emphasized the central role of antibiotic prophylaxis and evidence-based practice in SSI prevention research, underscoring their sustained relevance across multiple surgical disciplines.

Several additional studies have explored SSI prevention across diverse surgical settings, including guideline development, implementation strategies, bundled interventions, antimicrobial prophylaxis optimization, and infection surveillance in both high- and low-resource environments, reinforcing the multifactorial nature of SSI prevention [34-50].

Recent literature has also addressed SSI prevention in specific patient populations and procedures, including immunocompromised patients, hip replacement surgery, and neuromuscular spinal fusion, highlighting the importance of tailored preventive strategies in high-risk settings [51-53].

Limitations

This bibliometric review has limitations. First, the analysis was based only on the Web of Science database; therefore, relevant studies indexed in other databases or published in languages other than English may have been missed. Second, citation-based metrics tend to favor older publications, which may result in recently published studies - particularly those from 2020 onward - appearing less influential despite their quality and relevance.

Another limitation is that the analysis was restricted to the 50 most-cited publications. Focusing on highly cited studies allows the identification of influential landmark work; however, it may exclude newer or less-cited research that is also methodologically important. For this reason, our findings should be interpreted as reflecting citation impact rather than representing the entirety of the SSI prevention literature.

## Conclusions

This bibliometric analysis provides an overview of the most influential research on SSI prevention by examining the 50 most highly cited publications in the field. The findings show that SSI prevention research has been predominantly shaped by studies from general and orthopedic surgery and has relied largely on retrospective and observational study designs. Although research activity increased notably during the 2010s, particularly alongside the adoption of standardized preventive strategies, high-level randomized and implementation-focused studies remain underrepresented in the most influential literature.

These results highlight an important gap between the widespread clinical application of SSI prevention measures and the strength of the evidence supporting them. While commonly used strategies such as antibiotic prophylaxis, bundled interventions, and infection-control practices are supported by highly cited studies, there remains a clear need for more robust prospective research. Future studies should prioritize well-designed multicenter trials, focus on underrepresented surgical specialties, and include data from diverse healthcare settings to strengthen the evidence base and improve the global effectiveness of SSI prevention efforts.
